# Ketogenic Diet Consumption Inhibited Mitochondrial One-Carbon Metabolism

**DOI:** 10.3390/ijms23073650

**Published:** 2022-03-26

**Authors:** Fan-Yu Hsu, Jia-Ying Liou, Feng-Yao Tang, Nga-Lai Sou, Jian-Hau Peng, En-Pei Isabel Chiang

**Affiliations:** 1 Department of Food Science and Biotechnology, National Chung Hsing University, Taichung 402, Taiwan; fangyu1004@gmail.com (F.-Y.H.); iliujul12@gmail.com (J.-Y.L.); looksusan2013@gmail.com (N.-L.S.); jianhau.peng@gmail.com (J.-H.P.); 2 Biomedical Science Laboratory, Department of Nutrition, China Medical University, Taichung 402, Taiwan; vincenttang@mail.cmu.edu.tw; 3 Innovation and Development Center of Sustainable Agriculture (IDCSA), National Chung Hsing University, Taichung 402, Taiwan; 4 Ph.D. Program in Microbial Genomics, National Chung Hsing University, Taichung 402, Taiwan

**Keywords:** ketogenic diet, medium-chain triglycerides, one-carbon metabolism, stable isotopic labeling experiments, mitochondrial formate production, glycine cleavage system

## Abstract

Given the popularity of ketogenic diets, their potential long-term consequences deserve to be more carefully monitored. Mitochondrially derived formate has a critical role in mammalian one-carbon (1C) metabolism and development. The glycine cleavage system (GCS) accounts for another substantial source for mitochondrially derived 1C units. Objective: We investigated how the ketogenic state modulates mitochondrial formate generation and partitioning of 1C metabolic fluxes. Design: HepG2 cells treated with physiological doses (1 mM and 10 mM) of β-hydroxybutyrate (βHB) were utilized as the in vitro ketogenic model. Eight-week male C57BL/6JNarl mice received either a medium-chain fatty-acid-enriched ketogenic diet (MCT-KD) or a control diet AIN 93M for 8 weeks. Stable isotopic labeling experiments were conducted. Results and Conclusions: MCT-KD is effective in weight and fat loss. Deoxythymidine (dTMP) synthesis from the mitochondrial GCS-derived formate was significantly suppressed by βHB and consumption of MCT-KD. Consistently, plasma formate concentrations, as well as the metabolic fluxes from serine and glycine, were suppressed by MCT-KD. MCT-KD also decreased the fractional contribution of mitochondrially derived formate in methionine synthesis from serine. With the worldwide application, people and medical professionals should be more aware of the potential metabolic perturbations when practicing a long-term ketogenic diet.

## 1. Introduction

Ketogenic diets (KD) composed of high fat and insufficient carbohydrates that were originally used to treat refractory epilepsy have been found to have metabolic benefits; hence, KD has become a dietary strategy for weight control and energy balance regulation [1]. In the state of insufficient carbohydrates, metabolism in the body tends to oxidize fatty acids, and the generated acetyl-CoA is combined with oxaloacetate that enters the citric cycle to generate energy. When oxaloacetate is exhausted and not enough to maintain the balance of the citric cycle, the acetyl-CoA produced by fatty acid oxidation starts to produce ketone bodies as an energy source for extrahepatic tissues [2]. Acetoacetate and β-hydroxybutyrate (βHB) are two common forms of ketone bodies in the body, which can be converted into each other through βHB dehydrogenase. The conversion of βHB to acetoacetate reduces the reaction from nicotinamide adenine dinucleotide (NAD+) to reduced NAD+ (NADH). As ketone body metabolism consumes fewer NAD+ than glucose metabolism, KD may increase cellular NAD+/NADH ratio in certain tissues compared to a regular diet [3].

**Figure 1:**
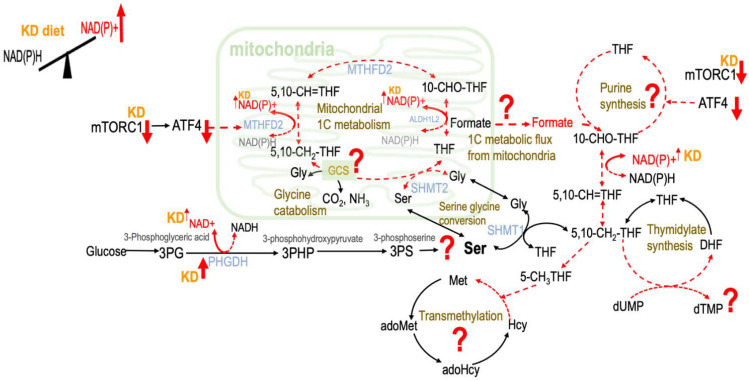
Proposed effects of ketogenesis on one-carbon metabolism. The conversion of β-hydroxybutyrate to acetoacetate reduces the reaction NAD+ to NADH, leading to increased NAD(P)+. KD—ketogenic diet; NAD+—nicotinamide adenine dinucleotide; NADP+—phosphorylated NAD+; NADH—reduced NAD+; NADPH—reduced NADP+; mTORC1—mechanistic target of rapamycin complex 1; ATF4—activating transcription factor 4; MTHFD2—the bifunctional methylenetetrahydrofolate dehydrogenase/cyclohydrolase; ALDH1L2—aldehyde dehydrogenase 1 family member L2; SHMT1—serine hydroxymethyltransferase 1; SHMT2—serine hydroxymethyltransferase 2; PHGDH—phosphoglycerate dehydrogenase; GCS—glycine cleavage system; THF—tetrahydrofolate; 5,10-CH_2_-THF—5,10-methylenetetrahydrofolate; 5,10-CH=THF—5,10-methenyltetrahydrofolate; 10-CHO-THF—10-Formyltetrahydrofolate; 5-CH_3_THF—5-methyltetrahydrofolate; DHF—dihydrofolate; dUMP—deoxyuridine monophosphate; dTMP—deoxythymidine monophosphate; Ser—serine; Gly—glycine; Met—methionine; Hcy—homocysteine; adoHcy—*S*-adenosyl-L-homocysteine; adoMet—*S*-adenosyl-L-methionine; 3PG—3-phosphserine.

The impact of ketogenesis on one-carbon (1C) metabolism is elusive and has not been carefully investigated. Ketogenesis was reported to inhibit mammalian target of rapamycin complex 1 (mTORC1) [4] to achieve life extension in mice. mTORC1 increases glycolytic genes glucose transporter 1 (GLUT1) and phosphofructokinase platelet isoform (PFKP) [5]. By the inhibition of the glucose uptake and glycolytic enzymes through mTORC1, ketogenesis may impede aerobic glycolysis and reduce 3-phosphoglycerate going toward the de novo serine synthesis pathway (SSP). On the other hand, ketogenesis may directly regulate SSP via the enzyme 3-phosphoglycerate dehydrogenase (PHGDH), the first step in the branching point from glycolysis to SSP. Ketogenic diets have been found to induce the expression of the SSP gene PHGDH in the liver and the cerebral cortex of mice [6], and such induction was proposed to assist the management of epilepsy by the ketogenic diet. The SSP contains an NAD+ dehydrogenase step catalyzed by PHGDH, and this pathway could contribute to NADH generation [7]; hence, we postulate that ketogenesis may modulate SSP (Figure 1).

The key 1C moiety carrier formate interconnects cellular and the whole body 1C metabolism. In vitro studies indicate that formate release requires active mitochondrial oxidative phosphorylation [8], and its production appeared to be favored when high concentrations of NADP+ and ADP were present [9]. Since ketogenesis has been reported to rapidly increase NAD+ levels in certain tissues [3], it is plausible that ketogenesis may modulate the partitioning of 1C metabolic fluxes via controlling formate release from the mitochondria by increasing the NAD(P)/NAD(P)H balance in vivo (Figure 1).

Mitochondrial glycine cleavage system (GCS) is a multienzyme complex that generates folate activated 1C units by catalyzing the reversible oxidation of glycine to carbon dioxide, ammonia, and 5,10-methylene tetrahydrofolate (5,10-CH_2_-THF) [10], which serves as an important route for the generation of 1C units [11]. Ketogenic diets have been reported to increase mouse muscle and liver AMP-activated kinase (AMPK) [1], which is known to increase intracellular NAD+, and NAD+/NADH ratio has been reported to increase in mouse brain during the ketogenic state. As hepatic glycine catabolism is regulated by the oxidation/reduction state of the mitochondrial NAD(H) and NADP(H) redox couples [12], it is plausible that ketogenesis also modulates glycine catabolism in the liver. We hypothesized that ketogenesis may impair mitochondrial GCS activity and limit the formate supply from the mitochondria in vivo. We aimed to establish model systems and trace the metabolic fate of mitochondrial GCS-derived formate during ketogenesis in vitro and in vivo.

In human 1C metabolism, the trifunctional mitochondrial enzyme methylenetetrahydrofolate dehydrogenase (MTHFD)/cyclohydrolase/10-formyl-THF synthetase is essential for the partitioning of 1C metabolism that is encoded by the MTHFD2 gene [13]. The expression of MTHFD2 was found to be regulated by the stimulation of activating transcription factor 4 (ATF4), which is activated by mTORC1 [14]. Many diets, including KD, that can prolong or regulate lifespan were proposed to achieve their effects by reducing mTORC1 activity [15]. A ketogenic diet was reported to reduce mTORC1 in rodent liver and the brain [4]. Based on this evidence, we speculated that ketogenesis may also modulate the partitioning of 1C metabolism by inhibition of MTHFD2 via suppression of mTOR activity (Figure 1).

Folate-mediated 1C metabolism is a major target of many therapies in human diseases [16,17,18,19]. Although several lines of evidence implied that ketogenesis may impact 1C metabolism, the impacts of ketogenesis and ketone bodies on 1C metabolic kinetics have not been fully illustrated. The current study systematically investigates whether ketogenesis may modulate glycolysis and SSP, mitochondrial formate release, glycine utilization and mitochondrial GCS, as well the partitioning of 1C metabolism (Figure 1). We predicted that the ketogenic state might impair formate generation and deplete formate supply that further interfere with 1C metabolism homeostasis. Combining an animal model and a cell model, we systematically explored whether KD may cause formate depletion and investigated its consequences on 1C metabolism.

## 2. Results

### 2.1. Consumption of MCT-KD Decreased Body Weight and Fat Mass but Increased Muscle Mass In Vivo

**Figure 2:**
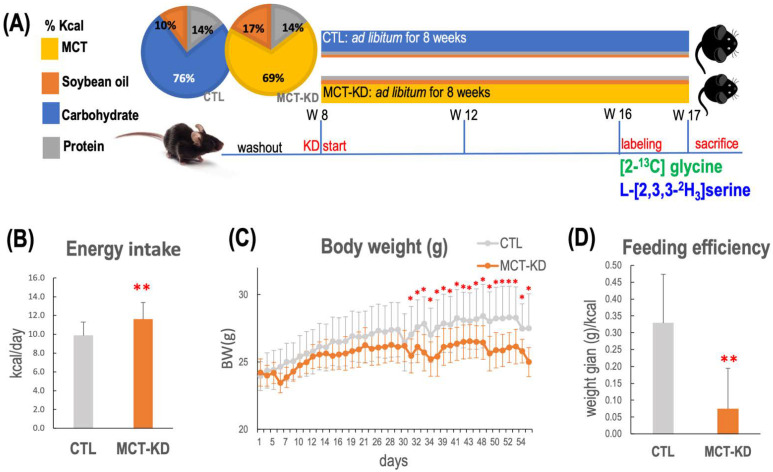
In vivo experimental design for investigation of ketogenesis on 1C metabolic fluxes. Body weight, energy intake, and feeding efficiency over the 8-week dietary intervention are shown. (**A**) Study design. After 1 week of habituation, mice were divided into 2 groups and fed with either a control diet (CTL) or a medium-chain enriched triglyceride ketogenic diet (MCT-KD) for 8 weeks. The 2 pie charts present the diet compositions of macronutrients. Ketone measurements were taken 4 weeks after diet intervention. (**B**) The mean energy intake of MCT-KD-fed mice was greater than that of the mice fed the (CTL) diet. (**C**) The body weight changes during the intervention period. The mean body weight of MCT-KD-fed mice became significantly lower 30 days after the initiation of the diet intervention. (**D**) The feeding efficiency was calculated as the total body mass gain divided by total kcal for each individual animal over the duration of the study. Feeding efficiency of MCT-KD was significantly lower than the CTL diet. For (**B**–**D**), data values represent group means (±SEM, MCT-KD, 12/group, * *p* < 0.05 vs. CTL, ** *p* < 0.01 vs. CTL).

Medium-chain fatty acids (MCFA) were used as the main fat sources in our ketogenic animal model [20]. All animals were fed designated diets *ad libitum* throughout the study (Figure 2A). Mice that received medium-chain triglyceride enriched ketogenic diet (MCT-KD) had significantly higher mean energy consumption (Figure 2B) (*p* < 0.0001, *n* = 12/group). Compared to the controls, the mean body weight of the MCT-KD mice became significantly lower 30 days after the initiation of MCT-KD intervention (Figure 2C). The feeding efficiency was significantly lower in MCT-KD mice (Figure 2D).

Plasma βHB concentration was used as an indicator for the ketogenic state. After 4 weeks of ketogenic diet consumption, although the changes appeared to be mild, plasma βHB concentrations modestly but significantly increased by 54% in mice fed MCT-KD compared to the controls (*p* = 0.009) (Table 1). The plasma βHB concentrations in this model indicated that 8-week consumption of our MCT-KD induced mild ketogenesis in healthy mice.

At the end of the study, the body weight of MCT-KD-fed mice was 8% lower than that of the controls (*p* = 0.014). The normalized (to body weight) weight of fat tissues including the brown adipose, the visceral adipose, and the adipose tissues around the reproductive organ significantly decreased by 25% (*p* = 0.035), 79% (*p* < 0.0001), and 61% (*p* < 0.0001), respectively, in MCT-KD compared to the controls. In contrast, the normalized muscle mass of MCT-KD mice significantly increased by 11% compared to the controls (Table 1).

### 2.2. Consumption of MCT-KD Decreased Plasma Formate Concentrations and Suppressed Formate Carbon Flow from Glycine and Serine

Plasma formate concentrations decreased by 19% in mice fed MCT-KD compared to the controls (Table 2). This observation supported our hypothesis that in vivo ketogenesis may suppress mitochondrial 1C metabolism. Plasma glycine concentration increased by 31% (Table 2), indicating that ketogenesis or MCT-KD may interfere with glycine utilization in vivo.

To further delineate the impacts of ketogenesis on mitochondrial 1C metabolism, [2-^13^C]glycine and [2,3,3-^2^H_3_]-Serine were used to trace the metabolic fate of mitochondrial folate metabolism, as serine and glycine are essential carbon sources that can produce formate via mitochondrial folate metabolism [21]. The plasma isotopic enrichments in formate + 1 significantly decreased by 38% from serine in mice fed MCT-KD. Moreover, formate + 1 enrichments from the glycine tracer were not detected in the plasma of MCT-KD-fed mice, indicating that MCT-KD strongly inhibited formate production via GCS (Table 2). These observations inspired us to further investigate the impacts of ketogenesis on GCS activity in vitro and in vivo.

### 2.3. β-Hydroxybutyrate Inhibited Mitochondrial Formate Generation from Glycine Cleavage System In Vitro

**Figure 3:**
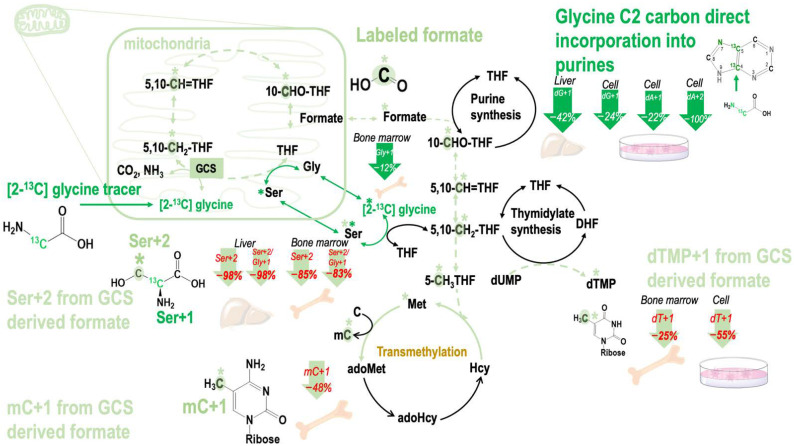
Effects of β-hydroxybutyrate (βHB) and MCT-KD consumption on the metabolic fate of glycine 2-carbon during glycine decarboxylation. The glycine cleavage system (GCS)-derived 1C moiety is exported from the mitochondria as formate. βHB inhibited mitochondrial formate generation from GCS in vitro. Ketogenic diet inhibited mitochondrial formate generation from GCS in mouse bone marrow. Abbreviations: GCS—glycine cleavage system; THF—tetrahydrofolate; 5,10-CH_2_-THF—5,10-methylenetetrahydrofolate; 5,10-CH=THF—5,10-methenyltetrahydrofolate; 10-CHO-THF—10-formyltetrahydrofolate; DHF—dihydrofolate; dUMP—deoxyuridine monophosphate; dTMP—deoxythymidine monophosphate; Ser—serine; Gly—glycine; Met—methionine; Hcy—homocysteine; adoHcy—*S*-adenosyl-L-homocysteine; adoMet—*S*-adenosyl-L-methionine; C—cytosine; mC—deoxy 5-methylcytosine; dA + 1—deoxyadenosine enrichments; dG + 1—deoxyguanosine; dTMP + 1—deoxythymidine; Ser + 1—serine enrichments; Gly + 1—glycine enrichments from [2-^13^C]glycine; Ser + 2—serine enrichments via mitochondria GCS.

Cells were treated with 1 mM and 10 mM βHB that reflected physiological ketone levels and the pathological conditions during ketosis [23] at the cell level, respectively. The isotopic enrichment in deoxythymidine synthesis (dTMP), dTMP + 1, decreased by 55% at 10 mM βHB in our cell model (Table 3A). These data indicated that GCS activity could be inhibited by βHB at the concentration of 10 mM. Enrichments from the original glycine tracer are marked in green, and enrichments from the recycled glycine 2-carbon from the GCS are marked in light green in Figure 3.

### 2.4. Consumption of MCT-KD Inhibited Mitochondrial Formate Generation from Glycine Cleavage System in Mouse Bone Marrow

The isotopic enrichments in dA + 1 from [2-^13^C]glycine did not differ between mice fed MCT-KD and the control diet (CTL) in the liver, except that the isotopic enrichments in dG + 1 tended to decrease (by 42%, *p* = 0.066). In bone marrow and liver proteins, the MCT-KD diet did not affect the serine enrichments Ser + 1 (Table 3B,C), indicating that MCT-KD did not affect the conversion between glycine and serine by cytosolic serine hydroxymethyltransferase (cSHMT). In the bone marrow, the MCT-KD diet tended to decrease glycine enrichments Gly + 1 but not Ser + 1. The isotopic enrichments in deoxyadenosine (dA + 1) and deoxyguanosine (dG + 1) did not differ between mice fed CTL and MCT-KD in the bone marrow (Table 3B).

In the liver, dTMP + 1 enrichments did not differ between mice fed CTL and MCT-KD. However, compared to the controls, MCT-KD drastically decreased Ser + 2 enrichments and the relative Ser + 2 enrichments from [2-^13^C]glycine by 98% (*p* = 0.031) and 98% (*p* = 0.012), respectively, in liver cellular proteins (Table 3C).

In the bone marrow, the isotopic enrichments in dTMP + 1 tended to decrease by 25% (*p* = 0.1) in the cellular proteins compared that of the controls (Table 3B). Moreover, compared to the controls, Ser + 2 significantly decreased by 85% (*p* = 0.045), and the relative enrichments of Ser + 2 decrease by 83% (*p* = 0.058) in the bone marrow cellular proteins of mice fed MCT-KD (Table 3B).

### 2.5. Effects of βHB on the Partitioning between Mitochondrial and Cytosolic 1C Metabolic Fluxes in Thymidine Biosynthesis In Vitro

In our cell model, treatment of βHB at 1 or 10 mM did not change dTMP + 1 enrichments. At 10 mM of βHB, dTMP + 2 enrichments tended to decrease by 7% (*p* = 0.1) (Table 4A), indicating that high βHB level may mildly affect utilization of cytosolic 1C utilization in dTMP synthesis to some extent. The total isotopic enrichments in dTMP did not differ between CTL and βHB treated cells. The fractional mitochondrial formate supply from the serine C-3 carbon (calculated from dTMP + 1 and dTMP + 2) remained unchanged after βHB treatments (Table 4A).

### 2.6. Consumption of MCT-KD Decreased Metabolic Fluxes from Mitochondrial Derived Formate In Vivo (Healthy Mouse Bone Marrow)

L-[2,3,3-^2^H_3_]serine was then used to trace the partitioning of serine 3-carbon between mitochondria and cytosol in dTMP synthesis in vivo. The fractional 1C fluxes from mitochondria were 90% in mouse bone marrow and 91% in mouse liver. These results matched well with the literature as well as our own previous studies [24], proving successful in vivo labeling was conducted.

In mouse bone marrow, consumption of MCT-KD decreased dTMP + 1 enrichments by 21% (*p* = 0.07), and it also decreased in dTMP + 2 enrichments by 25% (*p* = 0.06). The total enrichments of dTMP decreased by 22% (*p* = 0.07) compared that of the controls (Table 4B). The degree of reduction in the fluxes was similar between mitochondria and cytosol (Table 4B). These results indicated that consumption of MCT-KD suppressed the total utilization of 1C from serine in dTMP synthesis in both compartments. In mouse liver, MCT-KD decreased dTMP + 1 enrichments by 48% (*p* = 0.04) compared to that of the controls (Table 4C). Our results demonstrated that consumption of MCT-KD significantly suppressed the total utilization of 1C from serine in dTMP synthesis in bone marrow and liver without altering the partitioning of serine 3-carbon between mitochondria and cytosol, and the fractional carbon flow of mitochondrial 1C in mice undergoing mild ketogenesis remained similar to those in healthy mice.

### 2.7. Consumption of MCT-KD Altered Fractional Mitochondrial Formate Supply for Methionine Synthesis In Vivo

In the plasma-free amino acid pool, consumption of MCT-KD did not change the enrichments in Met + 1, Met + 2, the total of the two, or the fractional mitochondria-derived formate from L-[2,3,3-^2^H_3_]serine (Table 5A). In contrast, in mouse liver cellular proteins, MCT-KD decreased Met + 1 enrichments by 48% (*p* = 0.09); total enrichments in methionine tended to decrease by 35% (*p* = 0.1). MCT-KD decreased the fractional fluxes of mitochondria-derived formate by 23% (*p* = 0.05) (Table 5B). In contrast to the liver, consumption of MCT-KD did not change the enrichments in Met + 1, Met + 2, the total of the two, or the fractional mitochondria-derived formate from L-[2,3,3-^2^H_3_]serine in the bone marrow of mice consumed MCT-KD (Table 5A).

### 2.8. Consumption of MCT-KD Altered One-Carbon Metabolic Fluxes between 5-Methyl THF Dependent Methionine Synthesis and 5,10-Methylene THF Dependent dTMP Synthesis

In the plasma of MCT-KD-fed mice, the total leucine enrichments did not differ between MCT-KD mice and controls, indicating that tracer consumption was similar between the two groups. On the other hand, the total serine enrichments (Ser + 1 + 2 + 3) tended to increase by 91% (*p* = 0.1), particularly due to a trend of increased Ser + 1 (*p* = 0.1) and Ser + 2 enrichments (*p* = 0.1). These data indicated that mice fed MCT-KD may utilize serine differently from the controls. The glycine enrichments from L-[2,3,3-^2^H_3_]serine were investigated to trace serine and glycine conversion in cellular proteins. In liver proteins, total enrichments in leucine decreased by 30% (*p* = 0.1) and Gly + 1 enrichments decreased by 76% (*p* = 0.1). In bone marrow proteins, total enrichments in leucine decreased by 8% (*p* = 0.1) and Gly + 1 enrichments decreased by 27% (*p* = 0.1) (Table 6). These data indicated that consumption of MCT-KD may inhibit cSHMT activity.

Folate-dependent homocysteine remethylation (FDR) was calculated as the relative enrichments in methionine from L-[2,3,3-^2^H_3_]serine via 5-methylTHF. In plasma of mice consumed MCT-KD, FDR decreased by 23% compared to the controls (Table 7A). In the liver, FDR did not differ between MCT-KD and controls (Table 7B); total dTMP enrichments were minimal but were still found to be decreased in MCT-KD-fed mice (Table 7C). In the bone marrow, FDR significantly reduced in MCT-KD-fed mice (Table 7D). Then, the ratio between the total enrichments in methionine and dTMP was significantly lower in the MCT-KD-fed mouse bone marrow but not in the liver (Table 7F,G). These results suggest that bone marrow 1C metabolism is sensitive to MCT-KD, and when serine-dependent 1C metabolism was suppressed by MCT-KD, fluxes in methionine synthesis from serine reduced more compared to that in dTMP synthesis. More studies on the 1C dependent transmethylation pathways are underway.

## 3. Discussion

**Figure 4:**
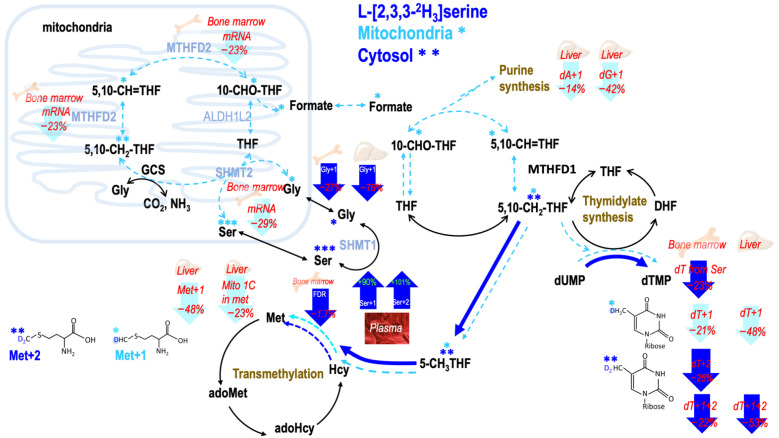
Tracing the partitioning of 1 carbon flux between mitochondrial and cytosolic derived one-carbon moiety via L-[2,3,3-^2^H_3_]serine. L-[2,3,3-^2^H_3_]serine tracer enables distinguishing mitochondrial (shown in cyan M + 1) derived 1C moiety from cytosolic (blue M + 2) counterpart. On the lower right, generation of de novo [^2^H_1_]-thymidine from mitochondria-derived formate via mitochondrial serine hydroxymethyl-transferase (mSHMT) and de novo [^2^H_2_] thymidine from cytosol via cytosolic SHMT (cSHMT). On the lower left, generation of [^2^H_1_] methionine from mitochondrial-derived formate and [^2^H_2_] methionine from cytosol via cSHMT. Enrichments in target metabolites were determined to investigate the impacts of ketogenetic state on formate utilization for thymidine and methionine syntheses from mitochondria (cyan M + 1) and cytosol (blue M + 2). Denotes the labeled metabolites. * ** *** Denotes M + 1, M + 2, M + 3, respectively. MTHFD2—the bifunctional methylenetetrahydrofolate dehydrogenase/cyclohydrolase; ALDH1L2—aldehyde dehydrogenase 1 family member L2; SHMT1—serine hydroxymethyltransferase 1; SHMT2—serine hydroxymethyltransferase 2; GCS—glycine cleavage system; THF—tetrahydrofolate; 5,10-CH2-THF—5,10-Methylenetetrahydrofolate; 5,10-CH=THF—5,10-Methenyltetrahydrofolate; 10-CHO-THF—10-Formyltetrahydrofolate; DHF—dihydrofolate; 5-CH_3_THF—5-methyltetrahydrofolate; dUMP—deoxyuridine monophosphate; dTMP—deoxythymidine monophosphate; Ser—serine; Gly—glycine; Met—methionine; Hcy—homocysteine; adoHcy—*S*-adenosyl-L-homocysteine; adoMet—*S*-adenosyl-L-methionine; dA + 1—deoxyadenosine enrichments; dG + 1—deoxyguanosine; Gly + 1—glycine enrichment; Met + 1, Met + 2—methionine enrichment from mitochondrial-derived formate and 5-CH_3_THF; dT + 1, dT + 2—deoxythymidine enrichment; dT + 1 + 2—deoxythymidine enrichment from both mitochondrial-derived formate and 5,10-CH_2_-THF.

The present study demonstrated novel findings that mild ketogenesis due to consumption of MCT-KD in healthy mice interferes with 1C metabolism in vivo, which include inhibition of mitochondrial formate generation from GCS (Figure 3), suppression of formate carbon flow from serine and glycine, and reduction in plasma formate concentration (Figure 3 and Figure 4).

### 3.1. MCT-Enriched Ketogenic Diet with Normal Protein Content Was Effective in Body Weight and Fat Mass Reduction

We chose (MCFA) as our major fat source as previous studies have shown that a diet rich in long-chain fatty acids (LCFA) may lead to obesity and insulin resistance and increase the risk of metabolic syndrome [25]. In contrast, MCFA has been reported to increase the oxidative capacity of muscles, prevent insulin resistance and metabolic syndrome, increase energy consumption, and reduce fat deposition in adipose tissues [26,27,28,29]. Moreover, our KD contained no carbohydrates. Mice fed a KD composed of 95% calories from fat, 0% from carbohydrate, 5% from protein was found to have a steady 15% weight loss compared to controls who consumed the same calories [1,30]. On the other hand, a previous study in mice fed a carbohydrate-free ketogenic diet, containing the macronutrient-calorie percent composition as 80% fat, 0% carbohydrate, and 20% protein, significantly increased the fat mass compared to the chow-fed control [31]. A previous study in rats suggested that the induction of ketosis in rodents is dependent on the high-fat content in combination with low protein content [32]. It has also been proposed that choline and methionine restriction, rather than carbohydrate restriction underlies many of the metabolic effects of KD [1,30]. These studies pointed out that macronutrient composition and protein content are both critical in generating a ketogenic state, and whether the KD-associated weight and fat loss are due to protein, choline, and methionine restriction was unknown.

Our MCT-KD diet with normal protein contents as the AIN93M diet (14% of calorie) successfully resulted in weight and fat loss, as well as improving muscle mass. These results demonstrated that an MCT-KD diet with no carbohydrate but normal protein is effective with respect to weight and fat loss that can also improve muscle mass, and protein/choline/methionine restriction in MCT-KD was not the main cause for weight and/or fat loss.

### 3.2. Ketogenesis Affects One-Carbon Metabolism in a Tissue-Specific Manner

Ketone bodies support bioenergetic homeostasis, particularly in the brain, heart, and skeletal muscle when carbohydrates are in short supply [33]. Alterations in the NAD+/NADH ratio by ketogenesis have been reported in the nervous system. Isolated mice neurons maintained in the presence of βHB exhibited increased oxygen consumption and ATP production and an elevated NAD+/NADH ratio [34]. Significant increases in hippocampal NAD+/NADH ratio and blood ketone bodies were detected only 2 days after and remained elevated at 3 weeks in rats fed ad libitum KD [3], indicating an early and persistent metabolic shift in the hippocampus. Blood βHB concentration reached 1 mM and was maintained at such level at week 3. The increased NAD+/NADH ratio has been verified in humans. The ratio of NAD+/NADH in the brain was increased in healthy humans reached nutritional ketosis [35]. On the other hand, the cortex NAD+/NADH ratio in rodents remained unchanged even 3 weeks after consumption of KD [3].

We speculated that increased NAD+ during ketogenic metabolism may be tissue/cell-specific, but less is known in other tissues regarding NAD+/NADH balance during the ketogenic state. Although increased NAD+ during ketolytic metabolism may be a primary mechanism behind the beneficial effects of KD, its impacts on 1C metabolism also remain to be elucidated.

In mammals, glucose, fatty acid oxidation, and ketone body metabolism all produce energy. In the nervous system, fatty acid oxidation is inefficient; hence, ketone bodies and glucose are the major energy sources. As ketone body metabolism consumes fewer NAD+ than glucose metabolism, ketogenic diets may increase the cellular NAD+/NADH ratio in the brain compared to a regular diet [3]. However, the impacts of NAD+/NADH balance from ketogenic diets on tissues outside the nervous system are less clear as other tissues also consume NAD+ through fatty acid oxidation to obtain energy. Consumption of a KD exerts highly tissue-specific effects, ultimately increasing mitochondrial turnover in the liver, while gene and protein expression in the brain remains tightly regulated. Ketogenic diets have been associated with the high activity of AMPK in mouse muscle and liver [4]. It has been demonstrated that AMPK enhances NAD+-dependent type III deacetylase Sirtuin 1 (SIRT1) activity by increasing cellular NAD+ levels [36]. These results suggest that ketogenesis may increase NAD+ by increasing AMPK in the liver and the skeletal muscle [36].

During ketosis, acetoacetate and βHB are interconverted by the mitochondrial enzyme β-hydroxybutyrate dehydrogenase (that oxidizes and reduces the mitochondrial NAD(H) redox couple) and are not metabolized further in the liver tissue. Accumulation of βHB during ketosis in the hepatic tissues may intensify the impacts of altered NAD+/NADH balance on 1C metabolism. Whether mild ketogenesis induced by consumption of the KD diet causes similar metabolic alterations is unknown.

### 3.3. Mild Ketogenic State Suppressed Mitochondrial Formate Supply in Liver and Bone Marrow via NAD+/NADP Balance

The concentration of blood ketone body in humans on a normal diet is 0.1 mM and is 7–8 mM in humans on a ketogenic diet [23]. Compared to humans on a ketogenic diet with a level of ketone body at 7–8 mM, the βHB level in our MCT-KD-fed mice was only mildly elevated, and the ketogenic state of our MCT-KD-fed mice was considered mild. Yet, the changes in 1C metabolism were significant and substantial. These findings raised concerns about humans who practice long-term ketogenic diets.

The effect of ketogenesis on 1C metabolism was elusive. Our MCT-KD diet successfully created a mild ketogenic state that significantly changed the utilization of serine, suppressed glycine utilization and mitochondrial GCS, reduced mitochondrial formate release, and changed the partitioning of 1C metabolic fluxes between dTMP and methionine synthesis.

Folate metabolism is known as a major source of NADPH for oxidative stress management [37]. It has been reported that a high cytosolic NADPH/NADP+ ratio favors flux from formate toward serine in the cytosol [38]. The key 1C moiety carrier formate interconnects cellular and the whole body 1C metabolism. It is also a central player between host and microbiome metabolism. The mitochondrial production of formate is essential for the endogenous generation of folate-related 1C moieties [39]. Mitochondria released formate is utilized to meet the cytosolic 1C demands for nucleotide [11,40] and methionine biosynthesis. Formate can also be used to re-synthesize serine via cytosolic 1C metabolism by cSHMT [11,41].

In vitro studies indicate that formate release requires active mitochondrial oxidative phosphorylation [8], and its production appeared to be favored when high concentrations of NADP+ and ADP were present [9]. Infusion of βHB into the liver of rats led to reducing the NAD(H) redox couple and inhibited the GCS [42], yet whether a mild ketogenic state also inhibits glycine catabolism in vivo has not been studied previously. In the present study, the consumption of KD-MCT inhibited the utilization of GCS-derived formate on synthesizing dTMP in the bone marrow and liver. Results from the current study supported our hypothesis that ketogenesis modulates 1C metabolism via NAD(P)/NADPH balance.

Mitochondrially derived formate has a critical role in mammalian metabolism and development. In humans, the GCS accounts for a substantial proportion of whole-body glycine flux. It was reported that glycine decarboxylation by the perfused liver was inhibited by 83% upon infusion of βHB [42]. Whether the impacts of KD on GCS differ between liver and extra hepatic tissues remained unknown. In mammals, glucose, fatty acid oxidation, and ketone body metabolism all produce energy. In the nervous system, fatty acid oxidation is inefficient; hence, ketone bodies and glucose are the major energy sources. On the other hand, the impacts of altered NAD+/NADP during ketogenesis on GCS in tissues outside the nervous system are complex, as other tissues can consume NAD+ through fatty acid oxidation to obtain energy.

In the isolated perfused rat liver, hepatic glycine catabolism was found to be regulated by the oxidation/reduction state of the mitochondrial NAD(H) and NADP(H) redox couples [12]. Our in vivo ketogenic model demonstrated that with very mild elevations in blood βHB, KD decreased the utilization of serine, suppressed glycine utilization and mitochondrial GCS, reduced mitochondrial formate release, and changed the partitioning of 1C metabolic fluxes between dTMP and methionine synthesis.

### 3.4. Ketogenic Diet May Exert Its Impacts on 1C Metabolism through Mitochondria Turnover

βHB has been shown to reduce the production of reactive oxygen species (ROS), improving mitochondrial respiration and bypassing the complex I dysfunction in mice brains [43]. βHB possesses an intrinsic high heat of combustion, making it an efficient mitochondrial fuel, where it can alter the NAD+/NADH and Q/QH_2_ couples and reduce the production of mitochondrial ROS [44]. Mitochondrion is a highly dynamic organelle that constantly fluctuates in morphology, biogenesis, and quality control in order to maintain cellular homeostasis. Consumption of KD decreased mitochondrial DNA, the amount of Complex II and Complex III proteins in the respiratory chain, as well as the total amount of mitochondria in mice liver [45].

Mitochondrial DNA depletion was found to induce an ATF4-mediated increase in serine biosynthesis and transsulfuration [46]. Lesioning the respiratory chain was also found to impair mitochondrial production of formate from serine. In some cells, respiratory chain inhibition leads to growth defects upon serine withdrawal, which are rescuable with purine or formate supplementation [46]. Experiments in isolated mitochondria indicated that formate produced by serine metabolism is respiration dependent [4]. In human HEK-293 cells, damaging the respiratory chain impaired the ability of serine metabolism to produce formate through the mitochondria [46]. These findings underscored the connection between the respiratory chain and 1C metabolism. Experiments in a diverse panel of proliferating cell lines [41] found that most 1C units are originated solely in the mitochondria [47]. By altering mitochondria turnover and respiratory chain, consumption of KD is plausible to change 1C metabolism. Our present study supported such a theory with the implications between mitochondrial pathogenesis during ketogenesis and perturbed 1C metabolism.

### 3.5. Ketogenic Diet May Exert Its Impacts on 1C Metabolism through mTOR Signaling

mTORC1 stimulates anabolism metabolisms such as de novo synthesis of proteins, nucleotides, and lipids in mammals [48]. Ketogenic diets have been associated with inhibition of the mTOR pathway in the mouse brain and liver [4], which could also be involved in the regulation of 1C metabolism. The inhibition of the mTOR signaling pathway by consumption of ketogenic diet in mice is most likely via the decreased Akt signaling in the brain and liver [4]. mTOR can increase the de novo synthesis of serine, thereby promoting one-carbon metabolism in mouse embryo fibroblasts [14]. Furthermore, the expression of methylenetetrahydrofolate dehydrogenase 2 (MTHFD2) was found to be regulated by the stimulation of activating ATF4 that is activated by mTORC1. mTORC1 has transcriptional effects on a variety of enzymes in purine synthesis that are also regulated by ATF4 [14]. mTORC1 assists the formation of 10-formyl-THF by promoting the expression of MTHFD2 and 10-formyl-THF that can provide a carbon source for purine synthesis [14]. Hence, we suggest that consumption of KD exerts its impact on 1C metabolism, at least in part, via mTOR signaling. On the other hand, since MTHFD2 expression and purine synthesis were stimulated by activating ATF4, one would expect to see inhibition of purine synthesis during ketogenesis. Interestingly, inhibition of purine synthesis was found only in cells treated with high βHB at 10 mM but not in 1 mM. The impact of KD consumption on the purine pathway in vivo was also quite mild, which is consistent with the only mildly increased blood βHB levels in our mouse model.

Taken together, consumption of ketogenic diet perturbs 1C metabolism possibly by alteration of NAD+/NADP balance, mitochondria turnover mTOR signaling pathway. Microarray profiling from mouse livers revealed that a significant subset of genes involved in 1C one-carbon metabolism was downregulated by consumption of KD [30], supporting our metabolic kinetic studies.

The main limitation of the present is that all observations were derived from cell and animal models from rodents. Future studies in human application are warranted.

## 4. Materials and Methods

### 4.1. Ketogenic Cell Model

All chemicals were purchased from Sigma-Aldrich (Sigma-Aldrich Inc, St. Louis, MO, USA) via the local distributor in Taiwan unless otherwise specified. To elucidate how ketogenic condition may affect cellular 1C metabolism, a stable cell-line established from the human hepatoma cell-line HepG2 that expresses human *glycine N-methyltransferase (GNMT)* [49] was used as a liver-derived cell model, as it is more relevant to normal hepatocyte metabolism compared to wild-type HepG2 cells and we have thoroughly characterized 1C metabolic kinetics [49] and it would be a better cell model for investigating hepatic metabolism compared to wild-type HepG2 cells [50].

Cells were cultured in α Minimum Essential Medium α (αMEM) with 10% fetal bovine serum (FBS), 1% Penicillin–Streptomycin–Amphotericin B Solution (PSA), at 37 °C in a 5% CO_2_ incubator. The ketogenic treatment was conducted as previously described [51]. In brief, 3 × 10^5^ cells were seeded in a 60 mm dish with α-MEM containing 10% fetal bovine serum. After seeding overnight, the medium was changed to one containing 10% fetal bovine serum, 1 mM or 10 mM DL-βHB sodium. Metabolic studies using isotopic tracers were conducted as described below.

### 4.2. In Vitro Isotopic Tracer Studies

To investigate the utilization of carbon C2-carbon of glycine, cells were cultured in minimum essential medium (MEM), supplemented with vitamin B12 alone with non-essential amino acid (NEAA), lipoic acid, pyruvate, ascorbate, serine (0.238 mM), and [2-^13^C]-Glycine (0.667 mM)(Cambridge Isotope Laboratories, Inc., Andover, MA, USA) [11]. The final media composition was comparable to that of the standard αMEM [11]. In the labeling experiments, isotopic enrichments from the tracers are calculated as molar ratios of labeled to non-labeled isotopomers after correction for the natural abundance of stable isotopes. The M + 1 is the isomer of the metabolite that contains 1 extra mass unit, and the M + 2 is the isomer of the metabolite that contains two extra mass units. The GCS-derived 1C moiety is exported from the mitochondria as formate. As glycine is decarboxylated and catabolized via the GCS in the mitochondria, the original ^13^C-labeled glycine 2-carbon is transferred to THF to yield ^13^C-labeled methyleneTHF, which ultimately produces ^13^C-labeled formate. Theoretically, this 1C unit can be utilized in cytosolic biosynthesis for nucleotides, serine, methionine, etc. [11]. After the labeling period, cells were collected, extracted [52], and derivatized for further analyses as previously described [53,54].

All experiments were performed in duplicates independently at least two times, and data from one experiment are presented. Isotopic enrichments in Ser + 2 and dTMP + 1 from [2-^13^C]glycine reflected the utilization of mitochondrial GCS derived formate in cytosol serine and dTMP synthesis [11].

### 4.3. Ketogenic Animal Model in Mice

**Figure 5:**
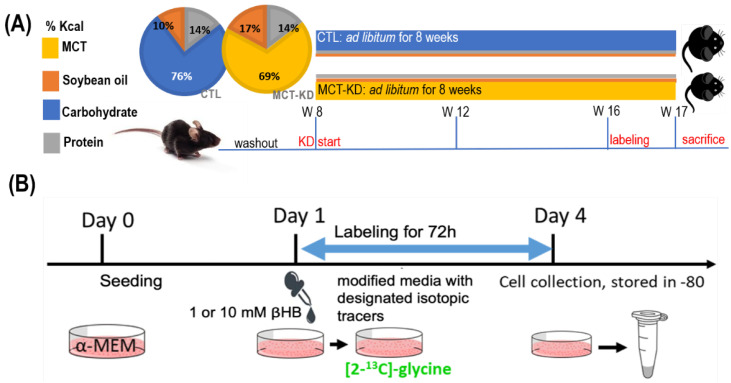
In vivo and in vitro experimental design. (**A**) Mouse model. After 1 week of habituation, mice were divided into 2 groups and fed with either a control diet (CTL) or a medium-chain enriched triglyceride ketogenic diet (MCT-KD) for 8 weeks (*n* = 8 vs. 12). The 2 pie charts present the diet compositions of macronutrients. (**B**) The cell model for investigating the effects of β-hydroxybutyrate on glycine catabolism and mitochondrial-derived formate utilization. Abbreviations: CTL—control groups; MCT-KD—medium-chain enriched triglyceride ketogenic diet; α-MEM—essential medium alpha medium; βHB—β-hydroxybutyrate.

The animal protocol was approved by the Institutional Animal Care and Use Committee of National Chung Hsing University. Twenty male 7 weeks old C57BL/6J mice were obtained from the National Laboratory Animal Center (NLAC, Taipei, Taiwan). After 1 week of habituation, mice were divided into two groups and fed with either a control diet (CTL) or a medium-chain enriched triglyceride ketogenic diet (MCT-KD) for 8 weeks (8 vs. 12). Mice were raised under specific pathogen-free conditions at 20–25 °C. The lighting was operated on a 12 h light-dark cycle. All mice had access to sterilized water *ad libitum*. Control mice were provided an AIN-93M diet (Dyets, Bethlehem, PA, USA). MCT-KD mice were provided with a homemade MCFA enriched ketogenic diet. The 2 pie charts in Figure 2A present the macronutrient compositions (Figure 5A), and the detailed components of the control diet and MCT-KD are shown in Table 8.

We chose MCFA instead of LCFA as our main fat source to avoid obesity, insulin resistance, or metabolic syndrome, and also for potential beneficial effects of MCT [26,27,28,29].

As for protein content, it has been proposed that protein level may have a major influence on mTORC1 signal in response to a KD [55], but further research is needed to understand better whether protein level is essential for ketogenesis and its related health benefits. We designed the MCT-KD with non-calorie from carbohydrate, as a previous study in mice fed a KD composed of 95% calories from fat without carbohydrate induced a steady 15% weight loss compared to controls consumed an isocaloric control diet [1,30]. We also made our diet with 14% calories from protein for the following reasons. The induction of ketosis in rodents was suggested to be dependent on the high-fat content in combination with low protein content [32]. However, whether low protein content is also essential for weight and fat loss is unknown. A previous study in mice fed a carbohydrate-free, normal/high protein ketogenic diet (80% of calories from lard and butter and 20% from protein) significantly increased the fat mass [31]. Therefore, we also aimed to examine whether KD diets with normal protein content can still successfully induce a ketogenic state and effectively result in weight and fat loss. The normal protein content in our MCT-KD also enabled us to examine whether the metabolic advantages induced by KD consumption are associated with methionine restriction. It has been proposed that choline and methionine restriction, rather than carbohydrate restriction, underlies many of the metabolic effects of KD [1,30]. With normal protein and choline contents in our MCT-KD, we successfully induced weight and fat loss. Finally, with normal dietary protein/amino acid contents, mice tissues could be successfully labeled with detectable enrichments in vivo using our labeling approaches.

Body weight was measured daily, and food intake was calculated weekly during the entire study. The feeding efficiency was calculated as the total body mass gain divided by the total kcal for each individual animal over the duration of the study. The mean body weight of MCT-KD-fed mice became significantly lower 30 days after the initiation of the diet intervention.

Ketone measurements were performed 4 weeks after the dietary intervention to check if mice reached a ketogenic state. After fasting for four hours, the mice were anesthetized with isoflurane, and the plasma was collected into a heparin coating tube through the orbital vein. The content of β-hydroxybutyrate in mouse plasma was determined by a β-Hydroxybutyrate Colorimetric Assay Kit (Cayman Chemical Company, Ann Arbor, MI, USA).

### 4.4. In Vivo Metabolic Tracer Studies

In vivo labeling studies were conducted to trace the metabolic fate of glycine 2-carbon and the partitioning of serine 3-carbon before the termination of the study. During this period, mice were fed a modified L-amino acid defined diet with the same amino acid composition as either the AIN-93M diet or the MCT-KD except for the substitution of designated tracer: [2-^13^C]glycine (for 144 h), or L-[2,3,3-^2^H_3_]serine (for 72 h) as described previously [11] (Figure 5B). The labeling diets were divided into small parts that were provided every 4 h during the labeling periods to ensure the tracers were slowly and evenly consumed during the labeling period [11].

In L-[2,3,3-^2^H_3_]serine experiments, methyleneTHF supplied by cSHMT and incorporated directly into methionine or dTMP retains the two deuterium atoms (CD_2_) on the hydroxymethyl group of serine. If the L-[2,3,3-^2^H_3_]serine enters the mitochondria and the hydroxymethyl group is released from the mitochondria as formate, it only contains a single deuterium atom (CD_1_) [56,57]. L-[2,3,3-^2^H_3_]serine was also used to trace folate-dependent homocysteine remethylation as described previously [24]. Folate-dependent homocysteine remethylation (FDR) was calculated as the relative enrichments in methionine from L-[2,3,3-^2^H_3_]serine [58].

Mice were sacrificed by isoflurane and cardiac puncture at the end of the labeling period after overnight fasting for 12 h. Blood was collected in heparin-coated tubes; the erythrocytes and plasma were separated immediately by 500× *g* centrifugation at 4 °C. Liver tissues were immediately excited upon sacrifice. Bone marrow cells were collected and washed by PBS. All tissues were stored at −80 °C until analysis as described [59].

### 4.5. Determination of Formate Concentrations and Enrichments from Glycine and Serine

Plasma formate concentrations were determined as previously described [60]. The isotopic enrichments in formate from [2-^13^C]glycine and L-[2,3,3-^2^H_3_]serine were calculated as molar ratios of labeled to non-labeled isotopomers after correction for the natural abundance of formate.

### 4.6. Determination of Amino Acid Concentrations and Enrichments from Glycine and Serine

Plasma and cytosolic free amino acids from cells or tissue samples were extracted using 0.4 M ice-cold perchloric acid (PCA) [59,61]. Cellular proteins were hydrolyzed in 6 N HCl under a vacuum. The amino acids were purified by cation-exchange chromatography, converted to heptafluorobutyryl-propyl ester derivatives, and separated on an HP-5MS column (30 m × 0.25 nm). Isotopic enrichment was determined in electron capture negative ionization mode by gas chromatography–mass spectrometry (GC-MS) using a model 6890 GC and model 5975C MS (Agilent, Palo Alto, CA, USA), as described previously [54,62].

### 4.7. Determination of Nucleotide Enrichments from Glycine and Serine

Genomic DNA was isolated and purified, as described previously [53]. The DNA samples were dried and hydrolyzed in formic acid under vacuum, then derivatized by N, O- Bis- [trimethylsilyl] trifluoroacetamide 1% trimethyl-chlorosilane and acetonitrile. Isotopic enrichments in deoxyadenylate (dAMP, dA), deoxyguanylate (dGMP, dG), deoxythymidine (dTMP, dT were determined in positive ionization mode by GC/MS, as described previously [53].

### 4.8. Statistical Analysis

Comparisons of the means between the control and the treatment groups were determined using the Student’s t-test. Results are expressed as mean ± SD. All statistical analyses were performed with SYSTAT 11.0 Windows TM (Systat software Inc., Richmond, CA, USA). For all analyses, the results were considered statistically significant if *p* values were <0.05. A trend of difference with a *p*-value < 0.1 is also reported.

## 5. Conclusions

In summary, we suggest that MCT-KD may not only reduce one-carbon metabolism through the mTOR pathway but also affect the supply of one-carbon metabolism in the mitochondria and even damage mitochondria.

Increased NAD during ketolytic metabolism may be a primary mechanism behind the beneficial effects of this metabolic therapy in a variety of brain disorders and in promoting health and longevity. However, there is a lot of evidence that the ketogenic diet has many health benefits, such as prevention of disease progression [63] and improvement in cognitive function [64]. Its impacts on 1C have not been investigated. We demonstrate for the first time that a ketogenic diet may suppress GCS and mitochondria 1C fluxes and re-direct the utilization and the partitioning of 1C metabolic fluxes in vivo. With the wide application and minimal restrictions on its implementation worldwide, people and medical professionals should be more aware of and be cautious of the potential metabolic perturbations when practicing a long-term ketogenic diet in humans. More metabolic studies on the ketogenic diet are warranted.

## Figures and Tables

**Table 1:** Effect of medium-chain triglyceride enriched ketogenic diet (MCT-KD) on body weight, body fats, muscle mass, and blood ketone concentrations ^1^.

	βHB ^3^ (mM)	BW ^3^ (g)	BAT/BW ^3^	VAT/BW ^3^	RAT/BW ^3^	Muscle/BW ^3^
CTL ^2^	0.37 ± 0.11	27.64 ± 2.3	0.003 ± 0.001	0.005 ± 0.003	0.02 ± 0.009	0.010 ± 0.001
MCT-KD ^2^	0.58 ± 0.16	25.48 ± 1.2	0.002 ± 0.000	0.001 ± 0.000	0.01 ± 0.002	0.011 ± 0.001
*p*-value	0.009	0.014	0.039	0.0001	0.001	0.017
% change	+54%	−8%	−25%	−79%	−61%	+11%

^1^ Values are presented as mean ± SD (CTL *n* = 8; MCT-KD *n* = 12). Data are calculated by Student’s *t*-test. ^2^ CTL—the control group; MCT-KD—MCT-enriched ketogenic diet group. ^3^ βHB—β-hydroxybutyrate; BW—body weight; BAT/BW—brown adipose tissue normalized to body weight; RAT/BW—reproductive adipose tissue normalized to body weight; VAT/BW—visceral adipose tissue normalized to body weight; muscle/BW—skeletal muscle from gastrocnemius muscle normalized to body weight.

**Table 2:** Plasma formate concentration and formate carbon flow from isotopic tracer L-[2,3,3-^2^H_3_]-Serine and [2-^13^C]-Glycine.

	Serine ^1^ (μM)	Glycine ^1^ (μM)	Formate ^1^ (μM)	Formate + 1 from L-[2,3,3-^2^H_3_]-Serine ^2^	Formate + 1 from [2-^13^C]-Glycine ^2^
CTL ^4^	180 ± 36	584 ± 149	27.0 ± 7.0	0.023 ± 0.002	0.002 ± 0.001
MCT-KD ^4^	214 ± 158	764 ± 244	21.7 ± 7.1	0.014 ± 0.001	Undetectable
% change ^3^		+31% ^#^	−19% ^#^	−38%	−100%

^1^ Plasma samples were extracted and analyzed for measuring the amino acid and formate concentrations (*n* = 8–12/group). These results are within mice’s normal range compared to the literature [22]. ^2^ Plasma formate enrichments in mice received L-[2,3,3-^2^H_3_]-Serine or [2-^13^C]-Glycine tracers (*n* = 2–3/group). ^3^ All values are presented as mean ± SD. Data are compared to the controls by Student’s *t*-test. The % change of each metabolite or the isotopic enrichments in each target compound was calculated by comparing it to that of the control group. Data with a % change shown in the table indicate statistically significantly compared to the controls (*p* < 0.05). Data with a % change shown with a *^#^* indicate a trend of difference compared to the control (*p* < 0.1). ^4^ CTL—the control group; MCT-KD—MCT-enriched ketogenic diet group.

**Table 3:** Ketone body and ketogenic state due to consumption of medium-chain triglyceride enriched ketogenic diet (MCT-KD) inhibited deoxythymidine synthesis (dTMP) derived from formate via mitochondrial glycine cleavage system in vitro and in vivo ^1^.

(A) BHB at 10 mM Inhibited dTMP Synthesis and Purine Synthesis from [2-^13^C]-Glycine In Vitro ^1^							
	From direct incorporation into DNA				From GCS derived formate		
	dA + 1 ^2^	dG + 1 ^2^		dA + 2 ^2^	dTMP + 1 ^2^		
CTL ^3^	0.323 ± 0.002	0.350 ± 0.003		0.011	0.027 ± 0.002		
BHB low ^3^	0.321 ± 0.010	0.340 ± 0.001		0.009	0.025 ± 0.001		
BHB high ^3^	0.253 ± 0.001	0.267 ± 0.018		0.000	0.012 ± 0.001		
% low BHB ^4^		−3% ^#^					
% high BHB ^4^	−22%	−24%%		−100	−55%		
(B) MCT-KD Inhibited the Utilization of GCS Derived Formate in Thymidine Synthesis in the Bone Marrow ^1^							
	From direct incorporation into protein/DNA				From GCS derived formate		
	Ser + 1 ^2^	Gly + 1 ^2^	dA + 1 ^2^	dG + 1 ^2^	dTMP + 1 ^2^	Ser + 2 ^2^	Ser + 2/Gly + 1 ^2^
CTL ^5^	0.046 ± 0.002	0.057 ± 0.001	0.085 ± 0.008	0.089 ± 0.013	0.011 ± 0.001	0.0008 ± 0.000	0.015 ± 0.003
MCT-KD ^5^	0.049 ± 0.003	0.051 ± 0.003 ^#^	0.072 ± 0.001	0.081 ± 0.001	0.008 ± 0.000	0.0001 ± 0.000	0.002 ± 0.001
% change ^4^	4%	−12% ^#^	−16%	−9% ^#^	−25% ^#^	−85%	−83% ^#^
(C) MCT-KD Inhibited the Utilization of GCS Derived Formate on Synthesizing Serine in the Liver ^1^							
	From direct incorporation into protein/DNA				From GCS derived formate		
	Ser + 1 ^2^	Gly + 1 ^2^		Ser + 1 ^2^	Gly + 1 ^2^		Ser + 1 ^2^
CTL ^5^	0.088 ± 0.005	0.070 ± 0.005	0.034 ± 0.005	0.058 ± 0.006	0.002 ± 0.000	0.001 ± 0.000	0.019 ± 0.002
MCT-KD ^5^	0.078 ± 0.008	0.061 ± 0.016	0.025 ± 0.001	0.034 ± 0.002	0.002 ± 0.000	undetectable	undetectable
% change ^4^	−11%	−13%	−25%	−42% ^#^	12%	−100%	−100%

^1^ Values are presented as mean ± SD (*n* = 2–3/group). Data are compared by Student’s *t*-test. ^2^ Ser + 1, serine enrichment; Gly + 1, glycine enrichment; dA + 1, deoxyadenosine enrichment; dG + 1, deoxyguanosine enrichment; dTMP + 1, deoxythymidine enrichment; Ser + 2, serine enrichment; Ser + 2/Gly + 1, the relative enrichment of serine from [2-^13^C]-Glycine. The enrichments in Ser + 1, Gly + 1, dA + 1, dG + 1, come from direct incorporation into protein/DNA. Enrichments in dTMP + 1 and Ser + 2 come from GCS derived formate from [2-^13^C]-Glycine. ^3^ CTL: control; BHB low: 1 mM β-Hydroxybutyrate dose in vitro; BHB high: 10 mM β-hydroxybutyrate dose in vitro. ^4^ The % change of the isotopic enrichments in each target compounds compared to the control group. Data with a % change shown in the table indicate statistically significantly compared to the controls (*p* < 0.05). Data with a % change shown with a ^#^ indicate a trend of difference compared to the control (*p* < 0.1). ^5^ CTL, controls; MCT-KD, MCT enriched ketogenic diet group.

**Table 4:** Partitioning between mitochondrial and cytosolic 1C metabolic fluxes in thymidine biosynthesis ^1,2^.

(A) Cell Model				
	dTMP + 1 ^3^	dTMP + 2 ^3^	Total ^3^	% Mito ^3^
CTL ^4^	0.295 ± 0.008	0.050 ± 0.000	0.345 ± 0.008	85%
βHB low ^4^	0.299 ± 0.012	0.049 ± 0.001	0.349 ± 0.012	86%
βHB high ^4^	0.296 ± 0.009	0.047 ± 0.001	0.343 ± 0.010	86%
% low βHB ^4^				
% high βHB ^4^		−7% ^#^		
(B) Mouse Bone Marrow DNA				
	dTMP + 1 ^3^	dTMP + 2 ^3^	Total ^3^	% Mito ^3^
CTL ^3^	0.086 ± 0.002	0.010 ± 0.00	0.096 ± 0.002	90%
MCT-KD ^3^	0.068 ± 0.005	0.008 ± 0.001	0.075 ± 0.005	90%
% change ^2^	−21% ^#^	−25% ^#^	−22% ^#^	
(C) Mouse Liver DNA				
	dTMP + 1 ^3^	dTMP + 2 ^3^	Total ^3^	% Mito ^3^
CTL ^4^	0.005 ± 0.000	0.0005 ± 0.000	0.005 ± 0.000	91%
MCT-KD ^4^	0.002 ± 0.000	0.0002 ± 0.000	0.003 ± 0.001	93%
% change ^2^	−48%	−55% ^#^	−49%	

^1^ Cellular DNA from cells and mouse tissues was extracted and analyzed for the deoxythymidylate (dTMP) synthesis using L-[2,3,3-^2^H_3_]serine as the tracer. ^2^ Values are presented as mean ± SD (*n* = 2–3/group) and compared by Student’s *t*-test. The % change of the isotopic enrichments in each target compound was calculated by comparing them to the control group. Data with a % change shown in the table indicate statistically significantly compared to the controls (*p* < 0.05). Data with a % change shown with a ^#^ indicate a trend of difference compared to the control (*p* < 0.1). ^3^ The dTMP + 1 enrichments from L-[2,3,3-^2^H_3_]serine reflected the 1C moiety from mitochondrial formate; the dTMP + 2 enrichments from L-[2,3,3-^2^H_3_]serine reflected the utilization of cytosolic 1C via cSHMT; Total, total enrichments of dTMP from both mitochondrial and cytosol formate; % Mito was calculated from dTMP + 1 and dTMP + 2 that represents the fractional mitochondrial formate supply for dTMP synthesis. ^4^ CTL—the control group; βHB low, cells were treated with 1 mM β-hydroxybutyrate; βHB high, treated with 10 mM β-hydroxybutyrate; MCT-KD—MCT-enriched ketogenic diet group.

**Table 5:** Fractional mitochondrial formate supply for methionine synthesis in vivo ^1,2^.

(A) in Plasma Free Amino Acid				
	Met + 1 ^3^	Met + 2 ^3^	Total	% Mito ^2^
CTL ^4^	0.011 ± 0.000	0.003 ± 0.000	0.016 ± 0.000	80% ± 2%
MCT-KD ^4^	0.013 ± 0.003	0.002 ± 0.002	0.014 ± 0.005	88% ± 12%
(B) in Liver Cellular Protein				
	Met + 1 ^3^	Met + 2 ^3^	Total	% Mito ^2^
CTL ^4^	0.012 ± 0.001	0.004 ± 0.000	0.017 ± 0.002	74% ± 1%
MCT-KD ^4^	0.006 ± 0.001	0.005 ± 0.002	0.011 ± 0.003	57% ± 4%
% change ^3^	−48% ^#^		−35% ^#^	−23%
(C) in Bone Marrow Cellular Protein				
	Met + 1 ^2^	Met + 2 ^2^	Total	% Mito
CTL ^4^	0.019 ± 0.001	0.007 ± 0.001	0.026 ± 0.002	75% ± 3%
MCT-KD ^4^	0.018 ± 0.001	0.006 ± 0.000	0.024 ± 0.002	75% ± 0%

^1^ Cellular proteins from mouse tissues were extracted and analyzed for the methionine synthesis using L-[2,3,3-^2^H_3_]serine as the tracer. ^2^ Values are presented as mean ± SD (*n* = 2–3/group) and compared by Student’s *t*-test. The % change of the isotopic enrichments in each target compound was calculated by comparing them to the control group. ^3^ Met + 1—methionine enrichment from mitochondrial formate; Met + 2—methionine enrichment using cytosolic one-carbon unit from L-[2,3,3-^2^H_3_]serine; Total—total enrichments of methionine from both mitochondrial and cytosol formate; % Mito was calculated from Met + 1 and Met + 2 that represents the fractional mitochondrial formate supply for methionine synthesis. Data with a % change shown in the table indicate statistically significantly compared to the controls (*p* < 0.05). Data with a % change shown with a ^#^ indicate a trend of difference compared to the control (*p* < 0.1). ^4^ CTL—the control group; MCT-KD—MCT-enriched ketogenic diet group.

**Table 6:** Utilization of L-[2,3,3-^2^H_3_]serine and serine to glycine conversion in vivo ^1,2^.

(A) Plasma						
	Leu + 1 + 2 + 3 ^1^	Ser + 1 + 2 + 3 ^3^	Ser + 1 ^3^	Ser + 2 ^3^	Ser + 3 ^3^	Gly + 1 ^3^
CTL ^4^	0.089 ± 0.000	0.042 ± 0.005	0.012 ± 0.002	0.020 ± 0.003	0.010 ± 0.001	0.003 ± 0.001
MCT-KD ^4^	0.089 ± 0.002	0.08 ± 0.019	0.022 ± 0.004	0.040 ± 0.009	0.017 ± 0.006	0.004 ± 0.001
% change ^3^		+91% ^#^	+90% ^#^	+101% ^#^		
(B) Liver Protein						
	Leu + 1 + 2 + 3 ^1^	Ser + 1 + 2 + 3 ^3^	Ser + 1 ^3^	Ser + 2 ^3^	Ser + 3 ^3^	Gly + 1 ^3^
CTL ^4^	0.099 ± 0.002	0.190 ± 0.035	0.046 ± 0.009	0.075 ± 0.003	0.069 ± 0.030	0.022 ± 0.006
MCT-KD ^4^	0.069 ± 0.007	0.143 ± 0.012	0.030 ± 0.007	0.067 ± 0.011	0.046 ± 0.006	0.005 ± 0.001
% change ^3^	−30% ^#^					−76% ^#^
(C) Bone Marrow Protein						
	Leu + 1 + 2 + 3 ^1^	Ser + 1 + 2 + 3 ^3^	Ser + 1 ^3^	Ser + 2 ^3^	Ser + 3 ^3^	Gly + 1 ^3^
CTL ^4^	0.110 ± 0.002	0.0196 ± 0.012	0.054 ± 0.003	0.073 ± 0.002	0.069 ± 0.006	0.039 ± 0.004
MCT-KD ^4^	0.101 ± 0.001	0.200 ± 0.018	0.050 ± 0.003	0.079 ± 0.008	0.070 ± 0.007	0.029 ± 0.003
% change ^3^	−8% ^#^					−27% ^#^

^1^ Mouse plasma and tissues were extracted and analyzed for the serine and glycine conversion and the isotopic distributions from [2,3,3-^2^H_3_]serine. Protein turnover was estimated by leucine tracer. ^2^ Values are presented as mean ± SD (*n* = 2–3/group) and compared by Student’s *t*-test. The % change of the isotopic enrichments in each target compound was calculated by comparing them to the control group. The % change is presented when values are statistically significant (bold) or with a trend (*p* < 0.1) of difference from controls (italic). ^3^ Putative isotopic enrichment of serine + 3: [2,3,3-^2^H_3_]serine (Ser + 3) is labeled with one deuterium atom at the C-2 and another two at C-3 position. The synthesis of 5,10-CH_2_THF acquires the methylene group (-CH2-) from serine, generating glycine. The deuterium atom in 5,10-CD_2_THF from cytosol via cSHMT and that from mitochondria-derived formate (CDOOH). In the reverse reaction, [2,3,3-^2^H_3_]serine can be synthesized from [2-^2^H_1_] glycine with the incorporation of the labeled methylene group of 5,10-CD2THF from cytosol. Putative isotopic enrichment of serine + 1: continued from above reaction, but in the reverse reaction, [2-^2^H_1_] serine (Ser + 1) is synthesized from [2-^2^H_1_] glycine with the incorporation of an unlabeled methylene group from 5,10-CH_2_THF, via cSHMT. Alternatively, [3-^2^H_1_]serine (Ser + 1) can be synthesized from unlabeled glycine with the incorporation of the labeled methylene group of 5,10-CDHTHF from mitochondria-derived formate. Gly + 1, glycine enrichment from L-[2,3,3-^2^H_3_]serine. Data with a % change shown with a ^#^ indicate a trend of difference compared to the control (*p* < 0.1). ^4^ CTL—the control group; MCT-KD—MCT-enriched ketogenic diet group.

**Table 7:** Partitioning of 5-methylTHF dependent methionine synthesis and 5,10 methylene THF dependent dTMP synthesis from L-[2,3,3-^2^H_3_]serine ^1,2^.

(A) The Methionine Synthesis from L-[2,3,3-^2^H_3_]serine in Plasma ^1^				
		Ser + 1 + 2 + 3 ^1^	Met + 1 + 2 ^3^	FDR ^3^
CTL ^5^		0.042 ± 0.005	0.016 ± 0.000	39.6 ± 4.9%
MCT-KD ^5^		0.08 ± 0.019	0.014 ± 0.005	16.3 ± 2.9%
% change ^3^		+91%		−23%
(B) Methionine Synthesis from L-[2,3,3-^2^H_3_]serine in Liver Cytoplasm				
		Ser + 1 + 2 + 3 ^4^	Met + 1 + 2 ^4^	FDR ^5^
CTL ^5^		0.149 ± 0.045	0.016 ± 0.006	10.9 ± 0.4%
MCT-KD ^5^		0.104 ± 0.013	0.017 ± 0.004	17.6 ± 5.7%
(C) Utilization of L-[2,3,3-^2^H_3_]serine in Liver Protein Methionine and DNA				
	via 5-methylTHF		via 5,10 methylene THF	
	Met + 1 + 2 ^4^	FDR ^5^	dT + 1 + 2 ^4^	dT from Ser ^4^
CTL ^5^	0.017 ± 0.002	9.2 ± 2.7%	0.0052 ± 0.000	2.9 ± 0.66%
MCT-KD ^5^	0.011 ± 0.003	7.8 ± 1.6%	0.0026 ± 0.001	1.8 ± 0.66%
% change ^3^	−32%	−16%	−53% ^#^	−34%
(D) Methionine Synthesis from L-[2,3,3-^2^H_3_]serine in Bone Marrow Cytoplasm				
		Ser + 1 + 2 + 3 ^4^	Met + 1 + 2 ^4^	FDR ^4^
CTL ^5^		0.196 ± 0.012	0.029 ± 0.009	41.5 ± 24.2%
MCT-KD ^5^		0.200 ± 0.018	0.017 ± 0.000	28.5 ± 2.1%
% change ^3^			−12% ^#^	
(E) Utilization of L-[2,3,3-^2^H_3_]serine in Marrow Protein and DNA				
	via 5-methylTHF		via 5,10 methylene THF	
	Met + 1 + 2 ^4^	FDR ^5^	dT + 1 + 2 ^4^	dT from Ser ^4^
CTL ^5^	0.023 ± 0.001	11.9 ± 0.2%	0.096 ± 0.002	122.8 ± 38.7%
MCT-KD ^5^	0.020 ± 0.001	10.2 ± 0.2%	0.075 ± 0.005	124.6 ± 18%
% change ^3^		−1.7%	−22% ^#^	
(F) Partitioning of 5,10 MethyleneTHF between Methionine and dTMP Thymidylate Synthesis in the Liver ^4^				
		mito 1C Met/dTMP ^4^	cyto 1C Met/dTMP ^4^	Total met/dTMP
CTL ^5^		2.593 ± 0.199	0.111 ± 0.002	0.316 ± 0.019
MCT-KD ^5^		2.723 ± 0.927	0.069 ± 0.069	0.270 ± 0.138
(G) Partitioning of 5,10 MethyleneTHF between Methionine and dTMP Thymidylate Synthesis in the Bone Marrow ^4^				
		mito 1C Met/dTMP ^4^	cyto 1C Met/dTMP ^4^	Total met/dTMP
CTL ^5^		0.238 ± 0.015	6.226 ± 4.018	4.143 ± 0.093
MCT-KD ^5^		0.232 ± 0.01	1.717 ± 0.255	3.717 ± 0.000

^1^ Mouse tissues were extracted and analyzed for the partitioning between 5-methylTHF-dependent methionine synthesis and 5,10 methylene THF-dependent dTMP synthesis from L-[2,3,3-^2^H_3_]serine. ^2^ Values are presented as mean ± SD (*n* = 2–3/group) and compared by Student’s *t*-test. The % change of the isotopic enrichments in each target compound was calculated by comparing them to the control group. The % change is presented when values are statistically significant (bold with *) or with a trend (*p* < 0.1) of difference from controls (italic with #). ^3^ Met + 1—methionine enrichment from mitochondrial formate; Met + 2—methionine enrichment using cytoplasmic one-carbon unit from L-[2,3,3-^2^H_3_]serine; Total—total enrichments of methionine from both mitochondrial and cytosol formate; % Mito was calculated from Met + 1 and Met + 2 that represents the fractional mitochondrial formate supply for methionine synthesis. FDR, folate-dependent remethylation of homocysteine, calculated by enrichments of methionine and serine. Data with a % change shown in the table indicate statistically significantly compared to the controls (*p* < 0.05). Data with a % change shown with a ^#^ indicate a trend of difference compared to the control (*p* < 0.1). ^4^ Partitioning of 5-methylTHF dependent methionine synthesis and 5,10 methylene THF dependent dTMP synthesis in mitochondria was calculated using dTMP + 1 and Met + 1; partitioning of 5-methylTHF dependent methionine synthesis and 5,10 methylene THF dependent dTMP synthesis in cytosol was calculated using dTMP + 2 and Met + 2. dT from Ser, the thymidine synthesis from serine was abbreviated as dT from Ser, and calculated as dT + 1 + 2/Ser + 1 + 2 + 3. ^5^ CTL—the control group; MCT-KD—MCT-enriched ketogenic diet group.

**Table 8:** Diet composition of MCT enriched ketogenic diet and control (AIN-93M).

Macronutrients	CTL (AIN-93M)	MCT-KD ^1^
Protein, % of energy	14.4%	14.4%
Carbohydrate, % of energy	75.4%	0.0%
Fat % of energy	10.2%	85.6%
Energy, kcal/g	3.60	5.89
Casein	140.0	140.0
L-Cysteine	1.8	1.8
Sucrose	100.0	0.0
Cornstarch	465.7	0.0
Dyetrose	155.0	0.0
Soybean oil	40.0	67.2
MCT oil	0	291.64
Cellulose	50	50
Salt Mix #210053	35	35
Vitamin Mix #310025	10	10
Choline bitartrate	2.5	2.5

^1^ CTL—the control diet; MCT-KD—MCT-enriched ketogenic diet.

## Data Availability

Not applicable.
